# News and Coming Events

**Published:** 1907-02-16

**Authors:** 


### NEWS AND COMING EVENTS.

THE Duke of Westminster has been re-elected President of the Chester General Infirmary.

A COMMITTEE has been appointed to make arrangements for suitably celebrating the hundredth anniversary of the founding of the Royal Halifax Infirmary.

DR. DONALD MACALISTER, Linacre Reader in Physic in the University of Oxford and President of the General Medical Council, has been elected Principal of the University of Glasgow.

DR. A. H. CARTER has resigned his position as senior physician to the Queen's Hospital, Birmingham, but will continue his duties as lecturer in medicine to the Birmingham University.

THE London Temperance Hospital, Queen Charlotte's Lying-in Hospital, and the Chelsea Hospital for Women have each received a present of twenty pheasants for the patients from his Majesty the King.

THE Committee of Management of the Radcliffe Infirmary, Oxford, has decided to appoint an honorary radiographer to the hospital. The appointment, which is for three years, is limited to qualified practitioners resident in Oxford.

MR. W. A. ADDINSELL, who has recently retired from the post of honorary secretary to the Birmingham Dental Hospital after nearly twenty-five years' active service, has been presented with several pieces of silver plate by the staff and subscribers to the hospital, as a token of their respect and regard.

THE following are the statistics for France given in a recently issued official publication :—

| | 1905. | 1904. | (Averages) 1894-1903. |
|---|---|---|---|
| Marriages ... | 308,623 | 290,721 | 292,718 |
| Divorces ... ... | 10,009 | 9,860 | 7,434 |
| Births ... ... | 807,291 | 818,229 | 846,246 |
| Deaths ... ... | 770,171 | 761,203 | 797,001 |

They show at a glance the significant decrease in the birth-rate, unaccompanied by a corresponding decrease in the rate of mortality.

THE annual report of Dr. Niven, medical officer of health for Manchester city, is more than usually interesting on account of the details given of the work done in Manchester in fighting consumption. One-third of all deaths between 15 and 35 years of age were due to phthisis, which is a notifiable disease in Manchester. Dr. Niven deals fully with the important subject of the treatment of discharged consumptives, and pleads for the establishment of a fund from which the family could be assisted "in case the bread-winner is struck down with the disease." He also details the notification scheme. The report is well worth reading by those who are interested in the question of consumption.

THE Pasteur Institute has benefited to the extent of nearly £1,000,000 under the will of a recently deceased French financier.

OBERLEHRER KAMMERER, a prominent representative of German missions, intends to start an institution in Germany on lines similar to those of the Livingstone College.

THE Milroy lectures on Kala-Azar and its epidemiology will be delivered by Major Leonard Rogers, I.M.S., at the Royal College of Physicians, Pall Mall East, on February 21, 26, and 28.

MR. SAMUEL ODAMES, one of Leicester's oldest inhabitants, has given £6,000 to build the first floor of a new wing to the local Infirmary, to be named the Samuel Odames Ward.

THE Hon. Secretary of the Clapham Dispensary states that the Hospital Fund have left the matter of uniform system of accounts for the dispensary to the Hospital Sunday and Saturday Funds.

WE have received the 18th edition of "Burdett's Hospitals and Charities." It contains informative chapters on the misuse of hospitals, hospital finance, and on the progress of hospitals in the United States, besides the usual details and hospital statistics.

THE Jewish Hospital at Leeds, founded and supported by Mr. Jacob Moser in memory of Dr. Herzl, the Zionist leader, has issued its first annual report, which shows that the institution is making satisfactory progress and that it undoubtedly justifies its existence.

ON Tuesday, February 19, the Therapeutical Society will hold its annual ladies' day and conversazione at Apothecaries' Hall, Blackfriars, E.C., at 4.30 P.M. There will be various exhibits, and Dr. Crichton will read a paper on the "Metric System in Prescribing and Dispensing."

BY the will of the late Mr. Richard Todd, of Blythewood, Edgbaston, nine charitable institutions in Birmingham are benefited. Legacies of £400 are left to the General and Queen's Hospitals, while the City Eye Hospital, the Children's Hospital, and the Moseley Convalescent Home receive £200 each. The orthopædic hospital and the deaf and dumb institution also get bequests.

THE collections on Hospital Sunday in Sheffield this year amounted to only £1,555, while ten years ago the sum raised was almost double. There has been a steady decrease in the collections. The local papers ascribe the smallness of the amount raised this year "to the fact that most people went skating that Sunday," and they question whether January is exactly the month for Hospital Sunday.

